# A Case of Artificial Anus Cured

**Published:** 1840-06

**Authors:** 


### CASE OF ARTIFICIAL ANUS CURED.

Dr. John Clark, of Morgan co., Ohio, has sent us the notes of a case of strangulated inguinal hernia, terminating in sphacelus and the formation of an artificial anus, which was promptly and effectually cured by using the forceps of Dupuytren. The patient, a female 50 years old, obstinately resisted the proposal to operate for the strangulation, and finally submitted to one still more distressing, for the depth and inflamed state of the walls of the great excavation almost rendered the proper application of the forceps impracticable. One resort to them proved to be sufficient. Their application was productive of a state of deep nervous prostration, which was relieved by stimulants. The Doctor informs us, that before the operation, alimentary matter, bile and fæcal matter, distinct from eaeh other flowed, at different times, through this artificial aperture. D.

